# A Nature-Inspired Approach to Energy-Efficient Relay Selection in Low-Power Wide-Area Networks (LPWAN)

**DOI:** 10.3390/s24113348

**Published:** 2024-05-23

**Authors:** Anna Strzoda, Krzysztof Grochla

**Affiliations:** Institute of Theoretical and Applied Informatics, Polish Academy of Science, Bałtycka 5, 44-100 Gliwice, Poland; kgrochla@iitis.pl

**Keywords:** LPWAN, LoRaWAN, 2-hop, relay device, sensor networks, nature-inspired algorithms, metaheuristics

## Abstract

Despite the ability of Low-Power Wide-Area Networks to offer extended range, they encounter challenges with coverage blind spots in the network. This article proposes an innovative energy-efficient and nature-inspired relay selection algorithm for LoRa-based LPWAN networks, serving as a solution for challenges related to poor signal range in areas with limited coverage. A swarm behavior-inspired approach is utilized to select the relays’ localization in the network, providing network energy efficiency and radio signal extension. These relays help to bridge communication gaps, significantly reducing the impact of coverage blind spots by forwarding signals from devices with poor direct connectivity with the gateway. The proposed algorithm considers critical factors for the LoRa standard, such as the Spreading Factor and device energy budget analysis. Simulation experiments validate the proposed scheme’s effectiveness in terms of energy efficiency under diverse multi-gateway (up to six gateways) network topology scenarios involving thousands of devices (1000–1500). Specifically, it is verified that the proposed approach outperforms a reference method in preventing battery depletion of the relays, which is vital for battery-powered IoT devices. Furthermore, the proposed heuristic method achieves over twice the speed of the exact method for some large-scale problems, with a negligible accuracy loss of less than 2%.

## 1. Introduction

Low-Power Wide-Area Networks (LPWANs) are a type of wireless telecommunication wide-area network characterized by long range, low cost, low throughput, and low power consumption. LPWAN technologies such as LoRa, NB-IoT, and Sigfox facilitate a vast range of IoT applications, from agricultural sensors and smart meters to asset tracking and smart city infrastructure, by providing cost-effective, efficient, and reliable connectivity solutions [1,2]. LoRa [3], developed by Semtech, is a prominent LPWAN technology that provides a higher data rate than SigFox and longer-range connectivity than the NB-IoT. Furthermore, LoRa operates on unlicensed frequencies (e.g., 868 MHz in the European Union, 902–928 MHz in the USA). LoRa utilizes Chirp Spread Spectrum (CSS) [4] modulation, a spread spectrum wideband technique that uses modulated linear frequency chirp pulses to encode information. This technology optimizes data transmission by adjusting the spreading factor (SF) parameter, e.g., from 7 to 12, balancing transmission speed, power usage, and operational range. This makes it well-suited for Internet of Things (IoT) and Smart City applications, particularly for telemetry and remote monitoring tasks.

Most LPWAN networks are based on a star topology, in which direct communication occurs between end devices and LoRa gateways. However, end devices in LoRa networks may have limited range, potentially leading to connection loss between nodes and gateways, especially in densely built or challenging terrain. In LoRaWAN, these issues are typically solved by adding more gateways, further complicating the system. Maintaining LPWAN gateways incurs significant cost due to their need for continuous connection to the central network server via the internet and their requirement to listen to all channels simultaneously, which elevates energy usage. Another approach involves enhancing the receiver’s sensitivity; although it improves signal detection, this results in a decreased data rate.

Utilizing relay devices provides a more effective and sustainable strategy for covering blind spots in the LoRa network. This solution extends signal range while minimizing network infrastructure deployment costs. The relay devices are battery-powered, and their hardware architecture closely resembles that of the end devices, as detailed in [5]. Installing relay devices that can serve as intermediaries by providing an additional communication hop between end devices and gateways emerges as a solution to enhance signal coverage within blind spots, thereby improving quality of service. The significance of the relay selection problem is gaining importance, as the role of relay devices in LoRa continues to be discussed in the scientific literature [6,7] as well as among standardization organizations. Since late 2022, the LoRa Alliance has published an extension to the LoRaWAN link layer specification introducing relay functionality [5]. However, while this specification introduces relay functionality, it does not address the placement of relay devices within the network to ensure operational efficiency, address coverage gaps, and minimize costs associated with network infrastructure development. Moreover, Semtech [8], a LoRa ecosystem provider, reports that strategic relay deployment is vital to solving problems with range in the LoRa network [9], highlighting the significance of the relay selection problem.

This paper proposes an energy-efficient relay device selection algorithm for LoRa networks. The paper is organized as follows: Section 2 provides a literature review; Section 3 details the heuristic relay selection algorithm; Section 4 provides an analysis of the parameters used in the proposed heuristic approach; Section 5 presents a performance evaluation in simulation environment; and a final discussion is provided in Section 6.

## 2. Literature Review

The concept of a relay node for the LoRa network was presented in [10]. This publication defined an extension of the LoRaWAN protocol, enabling end devices to detect and establish connections with a node that qualifies for the relay role. The described solution utilizes the Time-Division Multiple Access (TDMA) technique, constituting of an extension of the standard LoRaWAN protocol specification. The performance of the proposed solution was compared with the standard single-hop LoRaWAN communication scheme. The authors demonstrated the feasibility of using a battery-powered relay device in a LoRa network. The results of their experiments showed an increase in communication reliability and extension of the network’s range while maintaining the energy efficiency properties of LoRaWAN for relay-assisted communication. In [11,12,13], multi-hop communication schemes were considered using relay devices and an e-node to extend the range, aiming to enhance the reliability of transmitting data from LoRa devices to the gateway in IoT LoRa networks. In this context, Class C devices serve as relay devices, intercepting data transmissions from LoRa devices by eavesdropping and relaying them to the gateway. Additionally, each relay device alternately listens to transmissions from end devices in a receiving window and regularly forwards the content of intercepted packets to the gateway. However, these studies focused solely on increasing the probability of extending the range, overlooking the fairness principle of the transmission success probability for different network areas. In [14], the authors demonstrated the concept of a communication system considering the functionality of a relay node using available commercial components. Several earlier research works explored the relay selection problem in LPWANs and cellular networks.

In [7,15], the authors presented a relay node selection approach as a part of a system encompassing the optimization of resource allocation for the spreading factor, aiming to ensure maximum performance in terms of throughput, network coverage probability, and BER. The error rate performance is a relevant aspect for LoRa; thus, the authors of [16] presented new results for the BER performance of LoRa systems operating over various types of fading channels. Th simulation and numerical results in [7,15] demonstrated that such a system can improve BER and coverage probability for a given geographic area, but reduces throughput compared to the traditional LoRa system. Furthermore, the presented relay node selection technique did not consider analysis of the energy budget, which is crucial for battery-powered devices. Thus, energy efficiency is a key aspect and the subject of research in wireless sensor networks. The authors of [17] proposed a cascaded sensor dynamic activation and information fusion advanced mechanism which significantly improved the energy utilization efficiency and sensing performance of wireless sensor networks and has great potential for application.

In [18], the authors proposed an FSRC (Forwarding Signal with Relay Control) scheme for relay node management in IoT services within LPWAN networks based on LoRa technology. This scheme promotes the operation of relays with the involvement of end devices with a low spreading factor, aiming to maximize coverage and increase the transmission success probability for remote end devices. The proposed scheme considers a relay selection strategy based on the RSSI, theoretical values SNR, and Signal-to-Interference Ratio (SIR). Despite significantly increasing the packet delivery probability (by approximately 30%), the proposed approach does not account for the impact of relay functionality on the device’s battery life.

The approach presented in [19] serves as a benchmark for the SFPCR algorithm, demonstrating improvement in transmission success probability. The authors proposed a mechanism for addressing the “near–far fairness” problem in wireless networks, referring to the unequal quality of services depending on the distance to the base station. A technique based on network clustering into regions based on spreading factor coefficient values and a relay selection algorithm was applied to extend the network’s coverage and increase the transmission success probability. The relay selection algorithm is based on the harmonic mean of link quality and device energy resource indicators. The link quality was assessed based on the transmission success probability considering the distance between devices, SNR, and receiver sensitivity. The method was evaluated in the NS-3 simulation environment considering a LoRa network topology with only a single access point, whereas our approach is tailored for multi-gateway scenarios.

The in [20] presented an algorithm for selecting relays, wherein a single relay is assigned multiple weak nodes; however, such an approach may not be viable in systems where end devices transmit relatively frequently, such as parking, alarm, or security systems. Theoretically, an end device operating on SF = 7 could transmit dozens of times daily for up to ten years (including the duty cycle regulation). Managing multiple demanding weak devices with a single relay could lead to rapid battery depletion and frequent replacement of the relay device for weak nodes. In contrast, the solution that we propose assigns exactly one weak node to a single relay, which is a long-term and stable solution that makes the system more resilient and easier to manage.

In a well-known work, the authors of [21] proposed relay assignment optimization based on an auction model. The auction model provides incentives for partially cooperative users at the expense of introducing additional computational costs. The Energy-Efficient Maximum Weighted Matching (EE-MWM) algorithm was utilized for optimization. This approach is similar to the method presented in this paper, but does not consider energy consumption of devices.

In [22], the authors investigated relay-assisted device-to-device (D2D) communications for 5G wireless cellular networks. This approach, which focuses on relay selection with energy savings, introduces the PRS-D2D algorithm utilizing the Hungarian method [23] to solve the matching problem in polynomial time. It is important to note that this approach is not dedicated to the LoRa standard, and involves a configuration with directional antennas. The study in [24] explored the energy efficiency of LoRa across diverse topologies, encompassing both star and mesh networks. Their proposed strategy advocated the utilization of both star and mesh network topologies. The analysis considered the energy consumption of network density and range, employing various radio configurations; however, their paper did not delve into the selection of relay nodes to maximize the overall network throughput.

The authors of [25] demonstrated that relay selection algorithms in wireless networks can be executed with low computational complexity and system load. Their proposed semi-distributed user-relay algorithm does not exchange channel state information between network nodes; however, the algorithm was designed for IEEE 802.16 (WiMAX) networks, assuming very short distances between nodes (evaluated in a radius of 50 m), variable transmit power allocation (which is not typical in most LPWANs), and frequent communication between relays and the base station to exchange channel state information, which is not feasible in LPWANs due to very limited bandwidth.

Considering that the energy aspect often involves factors such as the SNR, RSSI, or distance between devices, above studies can help to simplify the assumption that a shorter distance implies lower energy consumption (though it should be noted that this is not always true due to obstacles, e.g., walls or buildings). There is a shortage of studies considering the device battery level, which is a critical factor for battery-powered IoT devices. Where such studies exist, they assign multiple weak nodes to a single relay, which is unsuitable for systems with relatively frequent transmissions (e.g., parking systems), as it overwhelms the relay with the energy demands of multiple connected devices. To fill this gap, we propose an approach that includes a comprehensive analysis of devices’ energy budgets and key LoRa parameters.

## 3. Relay Selection Algorithm

### 3.1. Introduction

LoRa network devices operate within a star topology wherein each device interacts with a LoRa gateway, facilitating straightforward and centralized communication. End devices may be installed in areas with limited coverage and may have poor connectivity to the gateway, resulting in the loss of some transmission packets and the need to assign a relay-type device. The role of the relay device is to forward signals from a end device with poor connectivity to the destination gateway.

The network topology is represented by an undirected graph $G=(V_{G},E_{G})$, with the set of nodes $V_{G}$ corresponding to network devices and set of edges $E_{G}$. The vertices are connected with an edge if corresponding devices are within range. Each edge $\left(v,w\right)\in E_{G}$ is assigned a weight $SF_{vw}$ representing a value of the spreading factor in the communication between the devices corresponding to nodes *v* and *w*.

#### 3.1.1. Problem Formulation

The problem is to find a set of devices that can serve as relays while identifying their operational zones to ensure full coverage for all given end devices with weak connections to the destination gateway (weak nodes) while also optimizing energy usage.

The relay selection problem in LoRa networks can be reduced to the problem of finding a matching with maximum weight among all maximum-cardinality matchings in a weighted graph. Its restriction to bipartite graphs is called the assignment problem, which is one of the classical combinatorial optimization problems [26]. This study considers two groups of nodes, namely, weak nodes and candidate nodes for relays, between which weighted edges define relationships. Thus, we consider an assignment problem wherein an assignment with the maximum total sum of weights should be found. This problem is a class P problem; algorithms such as Edmonds–Karp (EK) [27] or the Hungarian algorithm [23] (utilized in the reference method [21] used for performance evaluation Section 5) are known to solve it. However, we propose a heuristic approach based on Ant Colony Optimization (ACO) [28] that provides benefits related to the time required to obtain a solution with acceptable accuracy. The heuristic approach does not check all possible solution combinations; instead, it explores the solution space selectively. The heuristic function proposed in Section 3.3 is utilized in the exact EK method [27], with which the heuristic approach is then compared.

The weighted bipartite graph is the relay candidates graph $H=\left(U_{H},W_{H},E_{H},\eta \right)\subset G=\left(V_{G},E_{G}\right)$, where $U_{H}$ is a set of uncovered weak nodes (with the vertices corresponding to devices that need to be assigned to a relay), $W_{H}$ is a set of candidates for relay nodes, and $E_{H}$ is the set of edges between weak nodes and their neighboring candidates for relays, where each edge $\left(u,w\right)\in E_{H}$ is assigned a weight given by (12). This bipartite graph is an input for the proposed heuristic ACO approach and the exact EK algorithm.

##### Relay Node Selection with Constraints

For a given graph $H=(U_{H},W_{H},E_{H},\eta )$, we seek to find the set of a relay nodes $R\in W_{H}$ and the bipartite graph $J=(V_{J},E_{J})$ (which is the subgraph of H, J⊂H) satisfying the following conditions:

1. $V_{J}=U_{H}\cup R$
2. $E_{J}=\left\{\left(u,r\right):u\in U_{H}\wedge r\in R\wedge r\in N_{H}\left(u\right)\right\}$
3. $\forall u\in U_{H},\forall r\in R\deg_{J}\left(u\right)=deg_{J}\left(r\right)=1$
4. The sum of edge weights in graph *J* is the result of maximization:
  (1) $$\max\sum_{\left(u,w\right)\in E_{H}}\eta \left(u,w\right)x_{uw}$$
  (2) $$\sum_{u\in U_{H}}x_{uw}⩽1,\forall w\in W_{H}$$
  (3) $$\sum_{w\in W_{H}}x_{uw}=1,\forall u\in U_{H}$$
  where $x_{uw}\in \left\{0,1\right\}$ and $x_{uw}$ equals 1 denotes that the edge (u,w) is an edge in the weak node–relay assignment. If $|U_{H}\left|=\right|W_{H}|$, then $\sum_{u\in U_{H}}x_{uw}=1,\forall w\in W_{H}$ and $\sum_{w\in W_{H}}x_{uw}=1,\forall u\in U_{H}$.

### 3.2. Energy Consumption Model

In accordance with Casals et al. [29], the energy consumption involved in transmitting a LoRa packet can be segmented into distinct phases encompassing end device’s waking up, radio preparation, signal transmission, radio deactivation, and postprocessing. All of these phases except for signal transmission exhibit minimal or no dependency on resource allocation; hence, they are assumed to be uniform across all end devices in the proposed model.

Computational operations and costs associated with algorithms for selecting relays are deployed to run on a backend system (LoRa Network Server), not on the devices themselves. Offloading complex tasks to the backend system is common in LPWAN applications to keep the transceiver design simple and low-cost [30]; thus, these operations are not included in the energy consumption model concerning device battery usage.

The energy consumption of the end device during LoRa packet transmission and reception according to [8] is as follows:

(4) $$\begin{array}{c} T_{symbol}=\frac{2^{SF}}{BW} \end{array}$$

(5) $$\begin{array}{c} T_{preamble}=T_{symbol}\times \left(n_{preamble}+4.25\right) \end{array}$$

(6) $$\begin{array}{c} A=\left\lfloor \frac{8PL-4SF+28+16-20H}{4(SF-2DE)}\right\rfloor \end{array}$$

(7) $$\begin{array}{c} Payload=8+\max(A\times (CR+4),0) \end{array}$$

(8) $$\begin{array}{c} T_{payload}=Payload\times T_{symbol} \end{array}$$

(9) $$\begin{array}{c} T_{packet}=T_{preamble}+T_{payload} \end{array}$$

where: $T_{symbol}$—ToA for symbol, BW=125 kHz—bandwidth, $n_{preamble}=8$—number of symbols encoding the preamble (value specified in the “Regional Parameters LoRaWAN” [31]) and extended by an additional 4.25 symbols by the radio transmitter (resulting in 12.25 symbols), PL=51 B—payload size, H=0 B—header size, DE=1 if low data rate optimization is enabled (for SF = 11 and SF = 12), DE=0 for disabled, and CR=5—coding rate.

Then, the transmission energy usage $E_{TX}$ and reception energy usage $E_{RX}$ for a single packet are as follows.   

(10) $$E_{TX}=37\times T_{packet}$$

(11) $$E_{RX}=6.5\times T_{packet}$$

The calculation results for the presented energy consumption model are included in Table 1, and are utilized in the heuristic function described Section 3.3 and in the simulations in Section 5.

### 3.3. Heuristic Function

Each edge $\left\{u,w\right\}\in E_{H}$ is assigned a weight describing the quality of link between devices corresponding to nodes *u* and *w*. The formula for determining an edge weight is provided by the heuristic function $\eta :E_{H}\to R$,

(12) $$\eta \left(u,w\right)=\frac{E_{w}^{+}}{E_{RX_{u}}+E_{TX_{w}}},$$

where $E_{w}^{+}$ is the daily energy surplus (in mA) of the relay candidate *w*, calculated on the basis of the device’s battery level and remaining operation time [20]. The energy budget analysis includes the energy cost of switching to relay mode (1440 mAs). This value includes the cost of reconfiguring the settings and was established in collaboration with the industry partner while realizing the research grant nr POIR.04.01.04-00-0005/17. Nevertheless, our study is flexible in updating this variable. Here, $E_{RX_{u}}$ is the relay’s reception energy usage depending on the weak node’s *u* SF value, $E_{TX_{w}}$ is the relay’s transmission energy usage depending on the relay’s SF value, both of which are taken from Table 1.

The weight function (12) estimates the attractiveness of the connection between a weak node $u\in U_{H}$ and a relay node candidate $w\in W_{H}$. The Formula (12) consists of two main components: the energy surplus per day $E_{w}^{+}$ of the relay node candidate $w\in W_{H}$ (in the nominator), and the relaying energy cost of weak node $u\in U_{H}$ (in the denominator), which consists of the listening energy cost $E_{RX_{u}}$ and transmission energy cost $E_{TX_{w}}$ corresponding to a single packet of weak node $u\in U_{H}$ needing to be forwarded by a relay node *w*.

The heuristic function (12) considers a key parameter of LoRa technology, namely, the spreading factor SF∈{7,8,9,10,11,12}, which determines how many chirps (or symbols) are sent per second. Various spreading factor values result in significant differences in time-on-air (ToA) for a transmitting symbol [32]. With SF=n, a symbol can encode *n* information bits into a chirp, and the bit rate is provided by $R_{b}^{n}=n\cdot \frac{1}{2^{n}\;/\;BW}$, meaning that the symbol period is calculated by $T_{symbol}=\frac{2^{n}}{BW}$. Thus, when the number of bits in the symbol increases by only one, its ToA doubles; however, a higher SF means more resistance to interference and noise, resulting in a more extensive communication range.

In evaluating the link quality between a weak node and a relay candidate, it is necessary to consider two values of the SF, namely, the values for communications between a weak node and a relay candidate ($SF_{uw}$) and between a relay candidate and a LoRa gateway ($SF_{wg}$).

The impact of these SFs is captured in appropriate proportions in the denominator of the function (12). The formula considers the energy cost associated with the relay node’s listening, which is related to receiving packets from the weak node, and the energy cost of retransmitting those packets. It directly corresponds to the daily cost of serving as a relay for a weak node.

Nodes capable of serving as relays for individual weak nodes for as long as possible are prioritized, aiming to minimize future switches from relay mode.

The listening energy usage component $E_{RX_{u}}$ evaluates the node *w* based on the value of the spreading factor $SF_{uw}$ in the communication between a weak node *u* and the relay candidate *w*.

The transmission energy usage component $E_{TX_{w}}$ evaluates node *w* on the basis of the value of the spreading factor $SF_{wg}$ in the communication between node *w* and the destination gateway *g*. As the value of $SF_{wg}$ decreases, the value of the heuristic function (12) increases. This approach considers power-efficient relay node selection. The smaller the spreading factor to the destination LoRa gateway $SF_{wg}$, the less energy is used by the device represented by node *w* in relay mode. The higher the weight function value, the more attractive node $w\in W_{H}$ is to serve as a relay for weak node $u\in U_{H}$.

This clear principle, which divides the surplus energy of a relay node candidate by the cost of operating as a relay, precisely reflects the quality of connection from a weak node to the relay node candidate tp the LoRa gateway, while considering the role of node $w\in W_{H}$ as a relay for node $u\in U_{H}$.

The remaining operational time during which the device *w* must operate is already incorporated into the formula for determining the device’s energy surplus $E_{w}^{+}$.

**Figure 1:**
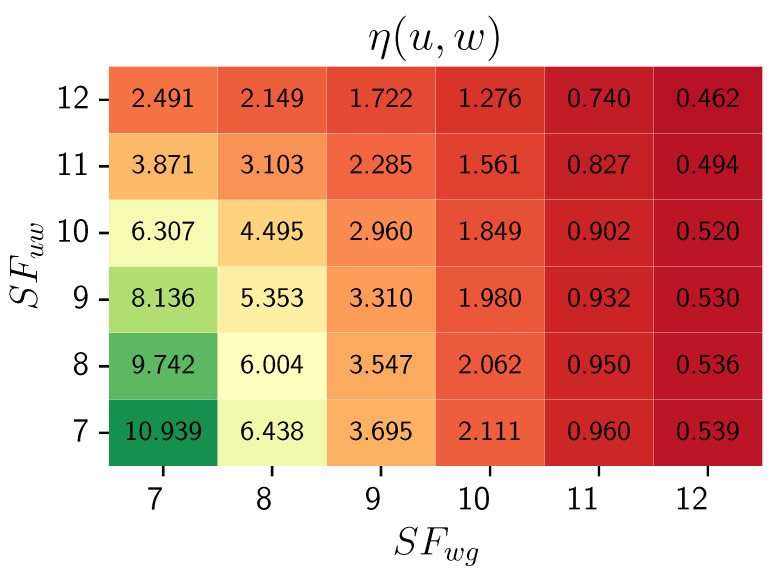
Edge weight function (12) distribution depending on SF parameters $SF_{uw}$ and $SF_{wg}$ for weak node $u\in U_{H}$ and relay candidate $w\in W_{H}$.

Figure 1 depicts the 2D distribution of weight function (12) values depending on the spreading factors in the communication between weak node–relay node candidate ($SF_{uw}$) and relay node candidate–LoRa gateway ($SF_{wg}$) for each $u\in U_{H}$, $w\in W_{H}$.

As the values of both spreading factors decrease, the edge weight function η(u,w) increases. The heatmap effectively highlights the differences in the objective function η(u,w) values for the cases $SF_{uw}=x_{1}$, $SF_{wg}=x_{2}$ and $SF_{uw}=x_{2}$, $SF_{ug}=x_{1}$, where $x_{1},x_{2}\in \left\{7,8,9,10,11,12\right\}\wedge x_{1}\neq x_{2}$. The SF in communication between the relay and the gateway ($SF_{wg}$) has a more significant impact on the relay’s energy consumption than the spreading factor in communication between the relay and a weak node ($SF_{uw}$). The heatmap aims to depict the characteristic influence of SFs and visualize their impact on outcomes. It is generated for a constant surplus energy value; the exact value is not significant, as the distribution characteristics are preserved.

To summarize the main characteristics of the heuristic function η (12) for providing an energy-efficient selection of relay-type devices, we emphasize the following:

- The heuristic function η promotes edges connecting potential relay nodes with a high energy surplus and a low maintenance cost for weak nodes. The value of η(u,w) increases as the numerator (energy surplus $E_{w}^{+}$) rises and the denominator (weak node maintenance cost $E_{RX_{u}}+E_{TX_{w}}$) decreases, enhancing the overall attractiveness of the relay node.
- The energy surplus $E_{w}^{+}$ is calculated based on the device’s battery level, a crucial feature for battery-powered IoT devices.
- The Spreading Factor, a vital transmission parameter in LoRa technology that influences battery consumption, is considered in the heuristic function η. The formula of η (12) appropriately accounts for this factor regarding the energy used to receive a packet from a weak node (component $E_{RX_{u}}$ calculated on (11)) and retransmit this packet (component $E_{TX_{w}}$ calculated on (10)).

### 3.4. Relay Selection Algorithm

Below is the pseudocode for the ACO relay selection algorithm. The result of Algorithm 1 corresponds directly to addressing the problem outlined in Section 3.1.1.

The probability of the *k*-th ant selecting node *w* at position *u* in the *i*-th iteration is formulated in [28] and provided by the formula

(13) $$p_{uw}^{k}\left(i\right)=\frac{{\left[\tau_{uw}\left(i\right)\right]}^{\alpha }{\left[\eta \left(u,w\right)\right]}^{\beta }}{\sum_{l\in N_{l}^{k}}{\left[\tau_{ul}\left(i\right)\right]}^{\alpha }{\left[\eta \left(u,l\right)\right]}^{\beta }},\forall l\in N_{u}^{k},\;$$

where the α and β parameters control the relative impact of the pheromone versus heuristic information η(u,w) (12) and $N_{l}^{k}$ is the set of the *k*-th ant’s unvisited adjacent vertices of node *u* at the *i*-th iteration.

The pheromone deposition function allows the algorithm to strive for better solutions in subsequent iterations, and is provided by the following formula [28]:

(14) $$\tau_{uw}\left(i+1\right)=\left(1-\rho \right)\tau_{uw}\left(i\right)+\sum_{k=1}^{m}\Delta \tau_{uw}^{k}\left(i\right)\;$$

(15) $$\Delta \tau_{uw}^{k}\left(i\right)=\left\{\begin{array}{c} \begin{array}{cc} \frac{\eta (u,w)}{Q}, & \left(u,w\right)\in M^{k}\left(i\right)\\ 0, & \left(u,w\right)\notin M^{k}\left(i\right) \end{array} \end{array}\right.$$

where *Q* is the constant value $max_{\left(u,w\right)\in E_{H}}\eta \left(u,w\right)$. This pheromone deposition strategy rewards edges based on their quality.
**Algorithm 1** ACO relay selection algorithm. Iterative approach finding a set of relays and their assignment to weak nodes. Takes a weighted bipartite graph $H=(U_{H},W_{H},E_{H},\eta )$ as input, where $U_{H}$ is a set of given weak nodes, $W_{H}$ is a set of candidates for relays, $E_{H}$ is a set of edges, η is the weight function of the edges, *t* is the number of iterations, and *m* is the number of ants. The procedure returns the best found assignment of relays to weak nodes.1:**function** aco_relay_selection($H=(U_{H},W_{H},E_{H},\eta )$, *t*, *m*)2:        $M_{best}\leftarrow \emptyset$▹ initialize result3:        $L_{best}\leftarrow -\infty$▹ initialize result’s weight4:        **for** i←1 to *t* **do**▹ for *i*-th iteration5:                paths←∅6:                **for** k←1 to *m* **do**▹ for *k*-th ant7:                      ${Avail}^{k}\left(U_{H}\right)\left(i\right)\leftarrow U_{H}$▹ initialize unvisited weak nodes8:                      ${Avail}^{k}\left(W_{H}\right)\left(i\right)\leftarrow {\left\{1\right\}}^{|W_{H}|}$▹ initialize unvisited potential relays9:                      $M^{k}\left(i\right)$, $L_{M}^{k}\left(i\right)$ = generate_ant_path(*H*, ${Avail}^{k}\left(U_{H}\right)\left(i\right)$, ${Avail}^{k}\left(W_{H}\right)\left(i\right)$) [Algorithm 2]10:                      $paths\leftarrow paths\cup \{M^{k}\left(i\right),L_{M}^{k}\left(i\right)\}$11:                      **if** $L_{M}^{k}\left(i\right)>L_{best}$ **then**12:                              $L_{best}=L_{M}^{k}\left(i\right)$13:                              $M_{best}=M^{k}\left(i\right)$▹ update best solution14:                **for** M∈paths **do**15:                      update pheromone decay based on (14) ▹ update pheromone decay16:        **return** $M_{best}$

**Algorithm 2** Procedure for ant path generation. Returns weak node–relay assignments and their respective weights as found by the *k*-th ant in the *i*-th iteration.1:**function** generate_ant_path($H=(U_{H},W_{H},E_{H},\eta )$, $Avail{\left(U_{H}\right)}^{k}\left(i\right)$, $Avail^{k}\left(W_{H}\right)\left(i\right)$)2:        $M^{k}\left(i\right)\leftarrow \emptyset$▹ initialize ant’s path3:        $L_{M}^{k}\left(i\right)\leftarrow 0$▹ initialize ant path’s weight4:        **while** $|Avail{\left(U_{H}\right)}^{k}\left(i\right)|>0$ **do**▹ while unvisited weak nodes exist5:                select $u\in {Avail}^{k}\left(U_{H}\right)\left(i\right)$▹ select random weak node6:                select node $w\in N_{u}^{k}\left(i\right)$ based on $p_{uw}^{k}$ (13)▹ select relay node7:                $M^{k}\left(i\right)\leftarrow M^{k}\left(i\right)\cup \left\{\left(u,w\right)\right\}$▹ add the assignment to the ant’s path8:                $L_{M}^{k}\left(i\right)=L_{M}^{k}\left(i\right)+\eta \left(u,w\right)$▹ update ant path’s weight9:                ${Avail}^{k}\left(U_{H}\right)\left(i\right)\leftarrow {Avail}^{k}\left(U_{H}\right)\left(i\right)\setminus \left\{u\right\}$▹ update unvisited weak nodes10:                $Avail^{k}\left(W_{H}\right)\left(i\right)\left[w\right]=0$▹ update available potential relays11:        **return** $M^{k}\left(i\right)$, $L_{M}^{k}\left(i\right)$▹ return the path and its weight

### 3.5. Computational Complexity

When establishing the running time of the ACO relay selection (Algorithm 1), the worst-case scenario was considered, i.e., a scenario in which the bipartite graph $H=(U_{H},W_{H},E_{H},\eta )$ is complete. In this analysis, the focus was on the most computationally significant components of the procedure, with the steps characterized as negligible being omitted.

In Table 2 and Table 3, the computational cost and the multiplicity of operations for Algorithm 1 necessary for determining the pessimistic execution time of the algorithm are presented. According to [33], each execution of the *k*th line requires time $c_{k}$, where $c_{k}$ is a constant.

Let $n_{1}=\left|U_{H}\right|$, $n_{2}=\left|W_{H}\right|$ and $n_{1}\leq n_{2}$.

Then, the pessimistic execution time of Algorithm 1 is presented as follows:

(16) $$T\left(n_{1},n_{2}\right)=t\cdot \left(m\cdot \left(c_{1}n_{1}+c_{2}n_{2}+c_{3}\sum_{j=1}^{n_{1}+1}j+c_{4}\sum_{j=1}^{n_{1}}\left(j+n_{2}\right)\right)+c_{5}n_{1}\right)\;$$

(17) $$\begin{array}{cc} \sum_{j=1}^{n_{1}+1}j & =\frac{\left(n_{1}+2\right)}{2}\left(n_{1}+1\right)≃n_{1}^{2}, \end{array}$$

(18) $$\begin{array}{cc} \sum_{j=1}^{n_{1}}\left(j+n_{2}\right) & =\sum_{j=1}^{n_{1}}j+\sum_{j=1}^{n_{1}}n_{2}=\frac{n_{1}+1}{2}n_{1}+n_{1}\cdot n_{2}, \end{array}$$

(19) $$\begin{array}{cc} \frac{n_{1}+1}{2}n_{1}+n_{1}\cdot n_{2} & ≃n_{1}^{2}+n_{1}\cdot n_{2}≃n_{1}\cdot n_{2} \end{array}$$

and, considering the asymptotic growth of the running time, the algorithm runs in time

(20) $$\begin{array}{cc} T(n_{1},n_{2}) & =O\left(t\cdot m\cdot n_{1}\cdot n_{2}\right)=O\left(t\cdot m\cdot U_{H}\cdot W_{H}\right)=O\left(t\cdot m\cdot E_{H}\right). \end{array}$$

In Table 4, the time complexity of the proposed methods (the heuristic ACO, the exact EK method [27], and reference method [21]) are compared.

Compared to the EK and reference methods, the computational complexity of the ACO algorithm does not solely depend on the size of the number of vertices and edges, as the number of iterations and number of ants involved are significant as well. The ACO algorithm is an alternative to the exact EK approach for certain problems. The proposed heuristic approach is suitable for large sparse graphs, i.e., where $|E_{H}|$ is significantly lower than $|V_{H}{|}^{2}$ [33]. ACO algorithm can find optimal or suboptimal solutions faster than exact methods for such cases, as presented in the subsequent Section 4.

## 4. Parameters Analysis

**Figure 2:**
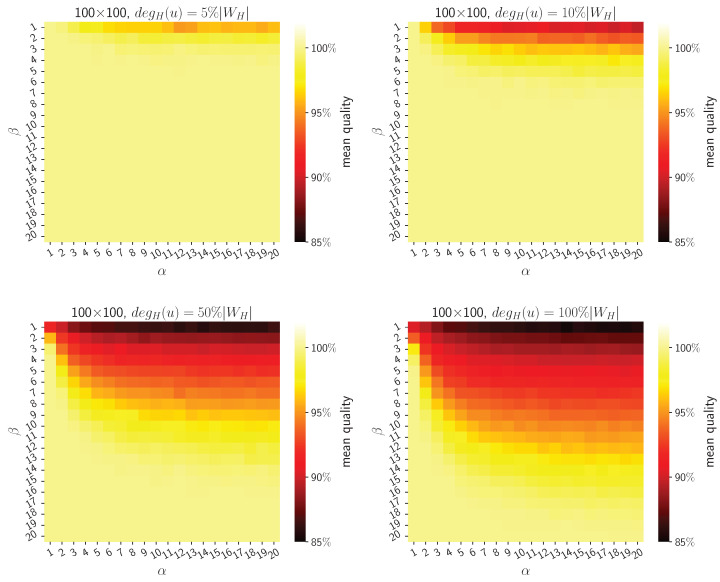
Distribution of ACO’s mean solution quality, depending on the problem’s size and method’s hyperparameters (α and β). Sparse and complete bipartite graphs are included in the results.

Figure 2 depicts the distribution of the mean quality for the ACO method depending on the problem size and values of the hyperparameters α and β. The mean quality value was obtained for 30 randomly generated bipartite test graphs, considering varying sparsity. The construction of the test graphs incorporated the specification that each graph must contain only one optimal solution, aiming for a precise assessment of algorithmic efficacy. The ACO method’s parameters were as follows. The total number of ants was equal to 20, as the ACO algorithm’s time complexity depends, among other factors, on the number of ants. As the number of ants increases, the algorithm’s runtime increases. Therefore, a huge number was not considered. The number of iterations was equal to 100. Notably, the algorithm does not always run for all iterations, as it implements an intelligent termination condition. The procedure terminates when an optimum solution is found (if any exists) or when the difference in solution quality remains less than or equal to $10^{-15}$ for 15 consecutive iterations (this value was selected experimentally). Using intelligent termination of the algorithm instead of executing an excessive number of iterations allows for faster achievement of results and avoidance of delay.

To estimate the amount of time saved, it is necessary to know the duration time of a single iteration. This time depends on the total number of ants *m* and the time needed for a single ant to generate a path, which depends on the problem size, e.g., the graph density as revealed in the theoretical analysis in Section 3.5.

The general formula for calculating the produced delay from extensive iterations can be formulated as follows:

(21) $$\left(t-t^{\prime }\right)\cdot m\cdot t_{path},$$

where *t* is the ACO algorithm hyperparameter, i.e., the total number of iterations, $t^{\prime }$ is the expected number of iterations needed to obtain acceptable accuracy of solution (which depends on the problem size), *m* is the total number of ants, and $t_{path}$ is the CPU time for generating single ant’s path (which also depends on the problem size.)

**Figure 3:**
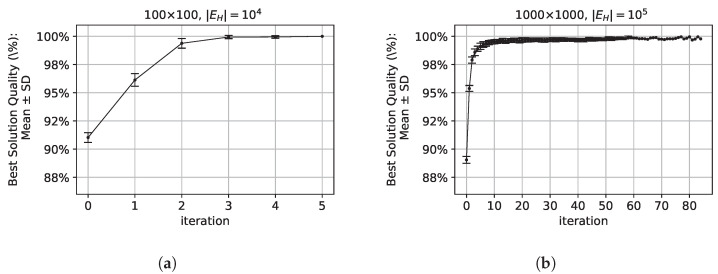
Convergence of the ACO algorithm for problems of varying sizes, specifically, the mean and standard deviations of the best solution at each iteration: (**a**) convergence of ACO for a relatively small complete graph (graph density 100%) $|U_{H}\left|=\right|W_{H}|=100$ and (**b**) convergence of ACO for a medium-sized sparse graph (graph density 10%) $|U_{H}\left|=\right|W_{H}|=1000$.

For the test case of a relatively small complete graph $|U_{H}\left|=\right|W_{H}|=100$, the algorithm finds the optimum solution up to the sixth iteration (Figure 3a). A tenfold increase in vertices and edges significantly extends the time needed to find the optimal solution, necessitating more algorithm iterations (Figure 3b). For the test case considered in Figure 3a, the CPU average time for executing a single iteration is on average 0.43(±0.03) s. For a graph with a tenfold increase in vertices and edges, which corresponds to relatively medium sparse graph (considered in Figure 3b), the CPU average time of a single iteration increases tenfold, i.e., 4.27(±0.15) s. Therefore, with a fixed number of iterations at t=100 and total number of ants m=20, without the intelligent termination conditions of an algorithm described in this work, the delay would average almost 40 s in the first case and approximately around 1 min in the second case.

The ACO algorithm’s behaviour, including its stability and convergence for problem instances of different sizes, are demonstrated in Figure 3. The figure presents the mean and standard deviations of the best solution in each iteration for a population of 30 random test graphs. These results illustrate the improvement in the solution with subsequent iterations, confirming the effectiveness of the pheromone deposition strategy (14), which enhances the solution in subsequent iterations. Furthermore, the decreasing standard deviation in the later iterations indicates a reduction in variation among the best solutions.

Apart from optical results, the AUC (Area Under the Curve) values are 4.9 for the curve in Figure 3a and 83.8 for the curve in Figure 3b (calculated for the mean solution quality values in range [0,1] and expressed as percentages in the figures). For both curves, the AUC quantity shows that the algorithm converges relatively quickly to an optimal or suboptimal solution. As each curve rises rapidly and reaches high values, the AUC value is close to the product of the optimal solution quality and the number of iterations (1×t, where 1 is the quality of optimum, and *t* is the number of iterations).

Table 5 presents the mean and standard deviations of the running time and accuracy for the heuristic ACO method and the exact EK method for relatively large problems, e.g., a graph with the total number of edges equal up to 10 million. The test cases represent the relay selection problem, considering a negligible ratio of weak nodes to candidate relay nodes, as the number of weak nodes in a properly designed network generally constitutes just a few percent of all devices. Additionally, weak nodes may be dispersed across the entire network, and may not share the same relay candidates within their range, making the test cases considered here sparse graphs.

The results indicate that the heuristic ACO method can operate more than twice as fast as the exact EK method while maintaining an accuracy of 98%. Therefore, for problems with such characteristics, the proposed ACO algorithm represents an alternative to the exact method.

## 5. Performance Evaluation

This section presents a performance evaluation of the proposed relay device selection algorithms in a simulation environment. The proposed heuristic ACO scheme is compared alongside the exact EK scheme and the well-known relay selection method [21], both of which are similar to the proposed approach. The evaluation used a simulation environment to operate LoRa networks with relay device functionality for various multi-gateway network topology scenarios involving thousands of nodes, as detailed in Section 5.2. A discrete event simulation environment, the Objective Modular Network Testbed in C++ (OMNeT++), was utilized to simulate the operation of the LoRa network. This model iteration builds upon the framework presented in a previous investigation [36], which concentrated on the collision probability derived from radio signal propagation models accessible in OMNeT++. In this study, the simulation model detailed in [20] is utilized.

### 5.1. Simulation Model

The simulation model’s architecture considers the operation of four categories of devices: the LoRa gateway, end devices, weak devices (end devices with poor direct connectivity with the gateway), and relay devices (devices that retransmit packets from the weak devices). This simulation model further implements functionality for computing the level of battery usage based on the energy model in Section 3.2 and the devices’ respective tasks. Each end device is configured to transmit one LoRa packet per day, while the relay devices receive and retransmit packets from weak devices. Battery level computations included the energy cost related to switching to relay mode. This cost is constant (1440 mAs) and subtracted once from the battery level of each device indicated to work in relay mode.

The key LoRa transmission parameters considered in the model are:

- Spreading Factor: determines a packet’s ToA, range, data rate, and energy consumption. The simulation model considered devices operating on different SFs (from 7 to 12).
- Transmission Power: refers to the power used by a transmitter to send signals (from −137 dBm to 14 dBm).

### 5.2. Simulation Scenarios

Diverse multi-gateway randomly generated network topology scenarios involving thousands of devices were utilized to evaluate methods through simulations. Table 6 presents the details of the network topologies scenarios, i.e., the total number of nodes, area, and percentage of weak nodes. Scenarios R(1000, 3) and R(1000, 5) account for over 600 nodes per 1 km^2^, while R(1500, 3) and R(1500, 5) feature around 160 nodes per 1 km^2^. Therefore, the topologies include areas with lower and higher node densities in space. Among the end nodes, percentages of 3% and 5% were randomly selected as weak nodes. Each network topology scenario included LoRa gateways selected through the procedure from [37]. This gateway selection method typically identifies more than one gateway for the considered network topology scenarios R(1500, 3) and R(1500, 5); on average, there are approximately six gateways (standard deviation 0.6), realizing a multi-gateway schema. Conversely, the algorithm typically identifies a single access point for smaller and denser topologies R(1000, 3), R(1000, 5).

#### 5.2.1. Experiment Scenarios

The experiments were divided into two parts, namely, an extensive and a demonstrative case, which were differentiated by the network devices’ initial battery levels. In the first group of experiments, all devices started with the same battery level. The second type of experiment was a demonstrative case involving a scenario where the devices’ initial battery levels varied. The extensive and demonstrative experiments are detailed below.

##### Extensive Case

The initial battery level for all network end devices was the same, allowing for transmission with the most pessimistic spreading factor of SF = 12 throughout the entire operational period of the network.

The study analyzed 30 random network topologies for each topology scenario listed in Table 6, totaling 120 network topologies. For each of these 30 topologies within every network topology scenario, three sets of relay nodes were selected using the ACO, EK, and reference [21] methods. Subsequently, simulations of each network’s operation over ten years were performed independently to measure the network-wide energy consumption for each of the three topologies. The simulation model incorporated various SF values on which the devices operated.

##### Demonstrative Case

The initial battery level differed for all network end devices depending on the SF value the device is configured to operate on throughout the entire operational period of the network. This strategy is related to the concept of manufacturers supplying devices adapted to specific areas. Devices set to operate on lower SFs consume less power; thus, equipping them with a battery sized for the highest SF can lead to energy wastage. Over time, this unused energy accumulates, resulting in unnecessary environmental and economic costs. Tailoring battery capacity to the specific operational SF of each device ensures more efficient use of resources. This enforces the device configuration for a designated SF and prevents wastage of excess energy allocated to devices that cannot consume it throughout their entire service life.

Each end device was assigned an initial battery level which representing the sum of the basic and surplus energy levels. The basic energy level (in mAs) was based on the SF parameter that the device is configured to operate on as well as on the simulation’s assumptions, such as transmitting one packet per day, network operation time, and the energy model described in Section 3.2. This basic energy level was estimated to ensure operation as a regular end device throughout the entire network operation period without the battery discharging. End devices were also granted a certain random extra energy drawn according to a uniform distribution [38] for each end device independently. Certain devices can use the additional energy surplus to act as relays; however, only some have enough surplus energy to perform this role.

The experiment involves one simulated 10-year period run for topology scenario R(1500, 3). The aim of this experiment was to illustrate the effectiveness of the proposed ACO energy-saving method for preventing battery depletion in comparison to the EK and reference methods [21].

#### 5.2.2. Results

Table 7 presents the results for the extensive case group of experiments. It involves simulations run side by side, differing in the set of relays selected for network 10-year operation time using the ACO, EK, and reference [21] methods.

Within a single topology scenario, differences in the average energy consumption in the network for various relay selection methods occur at $10^{-3}$. The experimental results show that the methods provide similar outcomes. The level of energy consumption in the network is similar, which proves that sets of relay devices selected independently by all methods are of similar quality.

**Figure 4:**
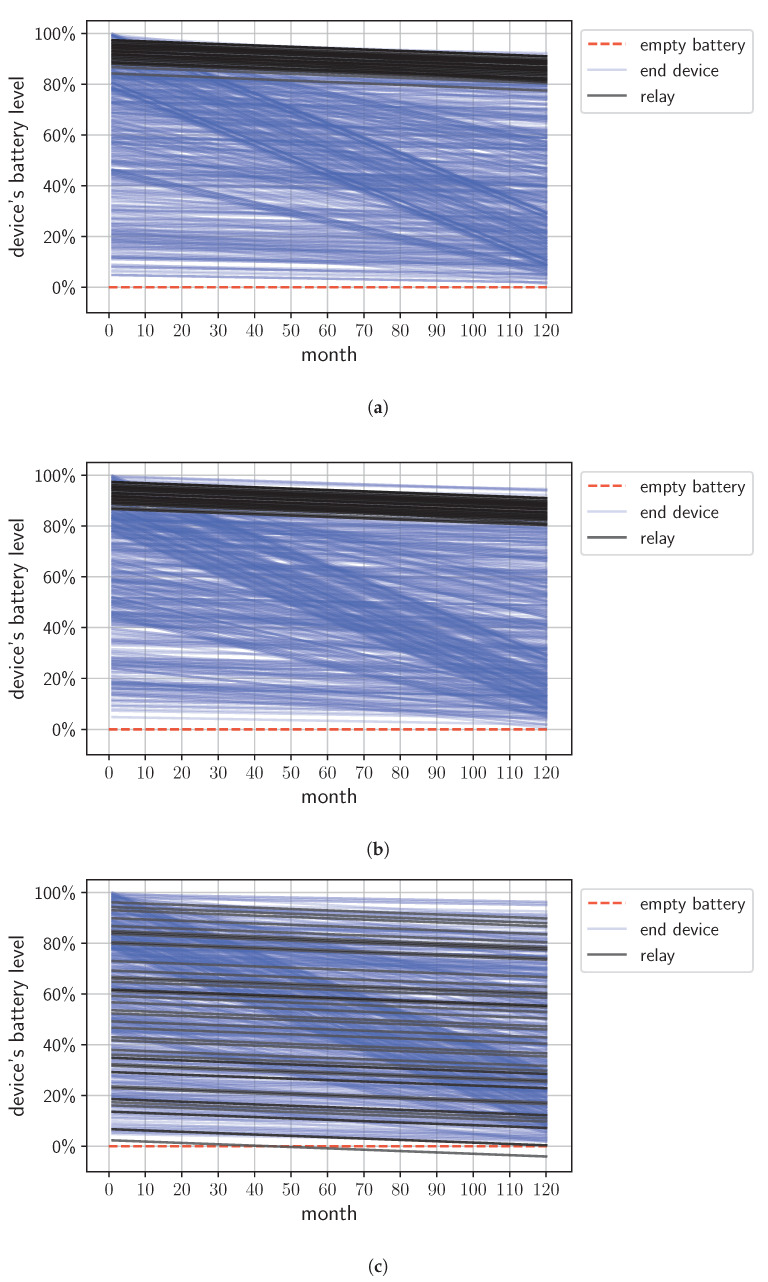
Change in device battery level consumption during the network’s operation over a 10-year period. The figures show the battery life for relay devices and other network end devices. (**a**) ACO relay selection method; (**b**) EK relay selection method; (**c**) reference relay selection method [21]. In the figure, the depletion of the battery of one of the selected relay devices is visible. The curve at the bottom of the chart that dips below zero in the 50th month corresponds to a relay device that has run out of battery.

However, the proposed ACO method and EK are particularly well adapted to LoRa technology; therefore, they are better optimized for this specific environment, especially for battery-powered devices. The proposed method allows for more effective network energy management by incorporating the devices’ energy consumption analysis. This aspect is crucial for battery-powered LoRa devices, as it prevents battery depletion and packet loss. Figure 4 depicts the simulation results for a demonstrative case utilizing the R(1500, 3) topology scenario. The figure specifically shows the change in the battery level of network devices operating over ten years for three independent simulation runs, each differing appropriately in terms of the set of relay-type devices selected by ACO, EK, and the reference [21] methods.

Curves corresponding to the relay devices are marked on the chart in different colors (black) than for the rest of the end devices. For devices receiving only a tiny energy surplus, their battery level would be near zero by the end of the network’s lifespan, as the initial battery level includes just enough charge to last ten years under typical end device operations. Nevertheless, utilizing the battery nearly to its total capacity is energy-efficient, as it prevents unnecessary surpluses which the device could not use during its operational period (most commonly set at ten years by the manufacturer [2]).

A device with an insufficient energy surplus cannot serve as a relay for the entire network operation duration, as it lacks the extra energy needed to listen to and retransmit additional packets from a weak device.

It can be observed that the resulting sets of relay nodes selected by the heuristic and exact methods are alike. The proposed ACO method and EK method prefer devices with a solid energy reserve, unlike the reference method, which does not consider energy budget analysis. The chart shows that the ACO and EK methods select nodes with high energy surplus as relays. The battery level curves for the group of relay nodes start from high values. The reference method [21] leads to battery depletion (indicated by a line dropping below zero), as shown in Figure 4c. An algorithm’s improper selection of relays, which are at risk of depleting their battery life, poses a significant issue. Such a scenario leads to the loss of data not only from the end node being served by the relay but also from the relay itself.

Furthermore, the ACO and EK methods promote nodes with a low SF relative to the access point as relay devices. This choice is evident from the gentle slope of the battery level change curves. A flatter slope indicates lower SFs in the communication between weak node, relay, and gateway, which is associated with the lowest energy consumption.

## 6. Discussion

This work proposes a novel nature-inspired energy-efficient relay selection scheme for LoRa-based LPWAN technology. It presents a heuristic approach based on Ant Colony Optimization alongside an exact algorithm, both innovatively designed with custom components for the specific challenges of relay selection in LoRa networks. It considers critical technological aspects such as SF and energy budget analysis, which are crucial for battery-powered IoT devices. Experimental results show that the heuristic approach operates up to twice as fast as the exact method, with a negligible accuracy loss of less than 2%, as shown on large-scale graphs (with up to one million edges). Therefore, the heuristic approach offers a flexible alternative to the exact method, especially for sizable sparse graphs.

Furthermore, comprehensive simulation experiments were conducted encompassing various scenarios within multi-gateway topologies consisting of thousands of devices operating across different spreading factors. Simulation results show that the proposed approach outperforms the well-known reference method in terms of providing to keep relay device batteries from running out. The proposed method is well-suited for high-demand deployments in which devices transmit signals relatively frequently. Assigning exactly one weak node to serve a single relay and promoting relays with significant energy surplus allows for better scalability and efficiency without overwhelming the network or causing contention with other nodes.

Finally, the proposed approach opens up new perspectives for further LoRa development, including network self-healing, the idea of a system that autonomously responds to faults or failures through dynamic switching devices to relay mode; moreover, the adaptability of this approach extends to network topology management in response to changes within its infrastructure, such as installing new devices. This adaptability facilitates seamless integration and optimization of network resources, ensuring robust and efficient connectivity even as the network evolves.

## 7. Conclusions

This study proposes a solution addressing relay selection challenges in LoRa-based LPWAN networks while considering crucial aspects such as energy budgeting, which is essential for battery-powered IoT devices. We present a heuristic approach as a more time-effective alternative to the exact algorithm for certain large-scale and sparse graph environments. The proposed energy-efficient approaches outperform traditional methods in preventing the depletion of device batteries, offering the potential for a self-healing concept in LoRa networks.

## Figures and Tables

**Table 1:** Energy consumption for a single packet’s transmission and reception depending on SF.

SF	Packet ToA [s] ($T_{\mathrm{packet}}$)	$E_{\mathrm{TX}}$ [mAs]	$E_{\mathrm{RX}}$ [mAs]
7	0.118	4.366	0.767
8	0.215	7.955	1.3975
9	0.39	14.43	2.535
10	0.698	25.826	4.537
11	1.56	57.72	10.14
12	2.796	103.452	18.174

**Table 2:** Computational cost and multiplicity of the significant components in Algorithm 1.

Component	Cost and Multiplicity
line 7: ${Avail}^{k}\left(U_{H}\right)\left(i\right)\leftarrow U_{H}$	$c_{1}\cdot n_{1}$
line 8: ${Avail}^{k}\left(W_{H}\right)\left(i\right)\leftarrow {\left\{1\right\}}^{|W_{H}|}$	$c_{2}\cdot n_{2}$
line 9: Algorithm 2	$c_{3}\cdot \sum_{j=1}^{n_{1}+1}j+c_{4}\cdot \sum_{j=1}^{n_{1}}\left(j+n_{2}\right)$ (based on Table 3)
line 15: update pheromone level based on [14]	$c_{5}\cdot n_{1}$

**Table 3:** Computational cost and multiplicity of the significant components in Algorithm 2 within a whole loop (4).

Component	Cost and Multiplicity
line 4: **while** $|Avail{\left(U_{H}\right)}^{k}\left(i\right)|>0$	$c_{3}\cdot \sum_{j=1}^{n_{1}+1}j$
line 6: choose vertex $w\in N_{u}^{k}\left(i\right)$ based on $p_{uw}^{k}$ [13]	$c_{6}\cdot \sum_{j=1}^{n_{1}}n_{2}$
line 9: ${Avail}^{k}\left(U_{H}\right)\left(i\right)\leftarrow {Avail}^{k}\left(U_{H}\right)\left(i\right)\setminus \left\{u\right\}$	$c_{7}\cdot \sum_{j=1}^{n_{1}}j$

**Table 4:** Computational complexity of the relay device selection methods, (heuristic ACO approach, exact EK approach [27], and reference approach [21]. Here, $V_{H}=U_{H}\cup W_{H}$.

Method	Computational Complexity
ACO	$O(t\cdot m\cdot E_{H})$
EK [27] ^1^	$O(V_{H}^{3})$
Reference [21] ^2^	$O(V_{H}^{3})$

^1^ Ref. [34], ^2^ Ref. [35].

**Table 5:** Average and standard deviations of the running time and accuracy of the heuristic ACO method versus the exact EK method for large sparse graphs.

$U_{H}\times W_{H}$	Graph Density	Avg. Time [s] ACO	Avg. Time [s] EK	ACO Avg. Time Savings	Avg. Accuracy ACO	Avg. Accuracy EK
$10^{3}\times 10^{4}$	5%	752 (±12)	1782 (±267)	58%	99% (±0.0002)	100%
	10%	1709 (±75)	3889 (±818)	56%	98% (±0.01)	100%
$10^{3}\times 10^{5}$	5%	12,971 (±897)	15,296 (±23)	15%	99% (±0.0009)	100%
	10%	29,556 (±1997)	41,935 (±9772)	30%	97% (±0.002)	100%

**Table 6:** LoRa network topology scenarios utilized in simulations.

Network Topology Scenario	Total Nbr. of Nodes	Area [m]	Weak Nodes
R(1000, 3)	1000	1000×1500	3%
R(1000, 5)	1000	1000×1500	5%
R(1500, 3)	1500	2500×3750	3%
R(1500, 5)	1500	2500×3750	5%

**Table 7:** Results of side-by-side runs of the proposed methods (ACO and EK) and reference [21] algorithm, specifically, the mean and standard deviations for the energy usage in the whole network with a set of relays selected by the three methods in each simulation scenario. The statistics were calculated for a population of 30 randomly generated network topologies.

Network Topology Scenario	Method	Mean Battery Usage (%)	Standard Deviation (%)
R(1000, 3)	EK	5.9550	1.2871
	ACO	5.9550	1.2870
	Referential [21]	5.9546	1.2868
R(1000, 5)	EK	6.2125	1.4176
	ACO	6.2125	1.4176
	Referential [21]	6.2123	1.4179
R(1500, 3)	EK	25.7492	0.7899
	ACO	25.7480	0.7894
	Referential [21]	25.7494	0.7898
R(1500, 5)	EK	25.4126	0.7799
	ACO	25.4122	0.7803
	Referential [21]	25.4180	0.7755

## Data Availability

All data available at the https://doi.org/10.5281/zenodo.11242472.
